# Pre-Meta: priors-augmented retrieval for LLM-based metadata generation

**DOI:** 10.1093/bioinformatics/btaf519

**Published:** 2025-09-18

**Authors:** Phil Tinn, Sondre Sørbø, Shanshan Jiang, Konstantinos Voutetakis, Sotiris Moudouris Giounis, Eleftherios Pilalis, Olga Papadodima, Dumitru Roman

**Affiliations:** SINTEF AS, Oslo 0373, Norway; SINTEF AS, Oslo 0373, Norway; SINTEF AS, Oslo 0373, Norway; Institute of Chemical Biology, National Hellenic Research Foundation, Athens 11635, Greece; Institute of Chemical Biology, National Hellenic Research Foundation, Athens 11635, Greece; Institute of Chemical Biology, National Hellenic Research Foundation, Athens 11635, Greece; e-NIOS Applications PC, Kallithea 17671, Greece; Institute of Chemical Biology, National Hellenic Research Foundation, Athens 11635, Greece; SINTEF AS, Oslo 0373, Norway; Bucharest University of Economic Studies, Bucharest 010374, Romania

## Abstract

**Motivation:**

While high-throughput sequencing technologies have dramatically accelerated genomic data generation, the manual processes required for dataset annotation and metadata creation impede the efficient discovery and publication of these resources across disparate public repositories. Large language models (LLMs) have the potential to streamline dataset profiling and discovery. However, their current limitations in generalizing across specialized knowledge domains, particularly in fields such as biomedical genomics, prevent them from fully realizing this potential. This article presents Pre-Meta, an LLM-agnostic and domain-independent data annotation pipeline with an enriched retrieval procedure that leverages related priors—such as pre-generated metadata tags and ontologies—as auxiliary information to improve the accuracy of automated metadata generation.

**Results:**

Validated using five selected metadata fields sampled across 1500 papers, the Pre-Meta assisted annotation experiment—without finetuning and prompt optimization—demonstrates a systemic improvement in the annotation task: shown through a 23%, 72%, and 75% accuracy gain from conventional RAG adoptions of GPT-4o mini, Llama 8B, and Mistral 7B respectively.

**Availability and implementation:**

The code, data access, and scripts are available at: https://github.com/SINTEF-SE/LLMDap.

## 1 Introduction

The sharing of data in biomedical and genomic research is essential for accelerating scientific discovery. The main challenge lies in effectively discovering, sharing, integrating, and analysing biomedical datasets in various isolated portals and databases (Byrd *et al.* 2020). The Research Data Repository Registry (https://www.re3data.org), a comprehensive global registry, lists more than 2000 repositories across various disciplines, with a significant portion dedicated to biomedical sciences. For example, the National Center for Biotechnology Information (NCBI) (https://www.ncbi.nlm.nih.gov) maintains several databases, including GenBank, which, as of 2023, contains more than two billion sequences. Similarly, NCBI’s Gene Expression Omnibus (GEO) archives gene expression data from over 100 000 experiments. The European Bioinformatics Institute (EBI) hosts ArrayExpress (https://www.ebi.ac.uk/biostudies/arrayexpress), including data from more than 70 000 assays. Collectively, these repositories house millions of datasets, each varying in size and complexity.

Published datasets are accompanied by metadata that provide descriptive information about the data. To enhance data discovery, it is crucial to annotate datasets with rich, high-quality metadata. However, fragmentation of data repositories creates significant obstacles for both data submission and discovery, which are crucial tasks in biomedical research. In data discovery, searching across multiple platforms is time-consuming and often results in incomplete or inconsistent findings due to differences in data formats, metadata descriptions, and access policies. In data submission, researchers face difficulties navigating the diverse requirements and formats imposed by different repositories when submitting datasets, thus necessitating extensive reformatting and manual curation, which increases the overall workload and risk of errors.

Large language models (LLMs), an increasingly powerful auto-regressive text generation technology based on the Transformer architecture (Vaswani *et al.* 2023), hold great potential in facilitating the processing and standardization of diverse metadata formats, thereby enhancing searchability and interoperability across disparate repositories. Retrieval-Augmented Generation (RAG) (Lewis *et al.* 2021) systems can further support discovery by retrieving relevant information from multiple sources and generating context-aware insights, minimizing the need for manual searches (Yang *et al.* 2025). In data submission, LLMs can assist researchers by automating metadata generation, ensuring adherence to repository requirements, and streamlining data formatting, ultimately reducing administrative burden.

Currently, several genomic data repositories are available, such as ArrayExpress (https://www.ebi.ac.uk/biostudies/arrayexpress), GEO (https://www.ncbi.nlm.nih.gov/geo), and cBioPortal (https://www.cbioportal.org). Each portal has its own defined metadatasets, but there is no harmonization across portals. For example, the metadata field of “study type” in ArrayExpress maps directly to “experiment type” in GEO, whereas cBioPortal uses fields such as “type of cancer”, “genetic alteration type”, and “study description” to define the characteristics of the dataset. Furthermore, repositories such as ArrayExpress and GEO differ in their metadata annotation schemas, with ArrayExpress employing the MAGE-TAB format (Brazma *et al.* 2001) and GEO utilizing the MINiML schema (Barrett et al. 2007), reflecting different priorities in metadata structuring. This heterogeneity means that data providers must use portal-specific metadata formats and follow distinct submission procedures, while data consumers must use different methods (e.g., APIs, filters, or interfaces) that may return inconsistent or incomplete results due to inadequate dataset annotation. As a result, researchers often resort to searching the literature for dataset discovery rather than relying on data portals, highlighting the urgent need for more structured and harmonized metadata annotation.

A reliable approach to addressing these inconsistencies is the use of biomedical ontologies, which provide structured vocabularies for dataset annotation in a standardized way. Ontologies such as the Ontology for Biomedical Investigations (OBI) (https://obi-ontology.org) and the Experimental Factor Ontology (EFO) (https://www.ebi.ac.uk/efo/index.html) define hierarchical relationships between biological concepts, enabling better metadata annotation and semantic interoperability across repositories. ArrayExpress, for instance, integrates EFO to ensure metadata consistency, while GEO aligns with NCBI’s ecosystem, incorporating structured metadata formats that facilitate machine-readability. Ontologies are also foundational in clinical and translational research, where resources such as SNOMED CT and MeSH support standardization in electronic health records (EHRs) and biomedical literature indexing (Bodenreider *et al.* 2018). Despite their transformative potential, biomedical ontologies are often underutilized in dataset annotation, limiting their impact on improving metadata discoverability and usability.

Recent advances in LLMs have demonstrated their ability to understand and generate structured and unstructured biomedical data, offering potential solutions to metadata fragmentation. However, LLMs are inherently biased due to their reliance on training data, which can be incomplete, imbalanced, or outdated (Thapa and Adhikari 2023). These biases manifest as an over-representation of well-studied diseases, datasets, and populations, while under-representing rare conditions, minority groups, and non-English sources (Nazi and Peng 2024). Furthermore, discrepancies in terminology, ontologies, and regional clinical practices often lead LLMs to propagate inconsistencies in metadata generation (Ayoub *et al.* 2024). This underscores the importance of integrating curated biomedical ontologies and leveraging domain-specific priors to improve the reliability of LLM-generated metadata annotations and dataset discovery.

Relevant methodologies can be inferred from the adjacent area of the Extreme Multi-label Classification (XMC) problem, which refers to a supervised learning setting in which a data point may be associated with multiple labels drawn from an extremely large label space (Bhatia *et al.* 2016). Relevant XMC works thus far have focused mostly on fine-tuning (Remy *et al.* 2022), prompt optimization (D’Oosterlinck *et al.* 2024), and multiplying the use of LLM calls (Zhu and Zamani 2023) to improve classification performance (further described in Section 4.4 Related Work); they have yet to be tested and extended to accommodate the diversity of data model standards seen across repositories and the heterogeneity of metadata fields inherent in genomic datasets.

To address these gaps, this research aims to leverage accessible structured data and Generative AI to provide rich, context-aware, and high-quality metadata annotation for biomedical datasets in an automated and efficient manner, supporting compliance with biomedical ontologies and repository/federating standards, such as Beacon V2 (Rueda *et al.* 2022) defined by the Global Alliance for Genomics and Health (GA4GH).

In this article, we introduce Pre-Meta, an LLM-based RAG pipeline for genomic metadata extraction that incorporates controlled vocabularies—in the form of pre-generated metadata and ontologies—as auxiliary data to improve annotation accuracy. Pre-Meta is model- and domain-agnostic, meaning that it provides a generic and adaptable metadata annotation procedure that is independent of both the LLM models used and the target metadata schema. We evaluated Pre-Meta using state-of-the-art open-weight and proprietary LLM models on real-world datasets curated from ArrayExpress and Europe PubMed Central (Europe PMC) and assessed its performance across five representative metadata fields. The results demonstrate that Pre-Meta significantly improves metadata annotation accuracy and highlight several systematic barriers that warrant further investigation. Furthermore, our work contributes to improving data stewardship promoted through FAIR principles (Findable, Accessible, Interoperable, Reusable) by reducing human intervention in the general process of data profiling, harmonization, and their downstream applications for data discovery.

## 2 Pre-Meta

### 2.1 Task definition

We define the task of data annotation as a process that, for a dataset or an asset of interest for (re)annotation, fills out a given form with metadata about the asset, using values that conform to the schema. The process takes as input:

- A textual description *x* of the asset, such as a published paper, research log, etc.
- A data model or schema *s* chosen among formats related to a data portal, sharing protocol, or newly defined metadata fields.
- Some auxiliary information or prior knowledge $z_{d}$ related to each metadata field *d* in schema *s*, such as a description, pre-generated metadata, or similar example values, e.g., from related ontologies.

The process can be represented as an RAG function f(·) that predicts each metadata label $y_{d}$ as an annotation to the target asset based on its textual description *x*:


(1) $${\hat{y}}_{d}=f(x,z_{d}),\;\forall \;d\in s$$

### 2.2 Implementation

**Figure 1.**
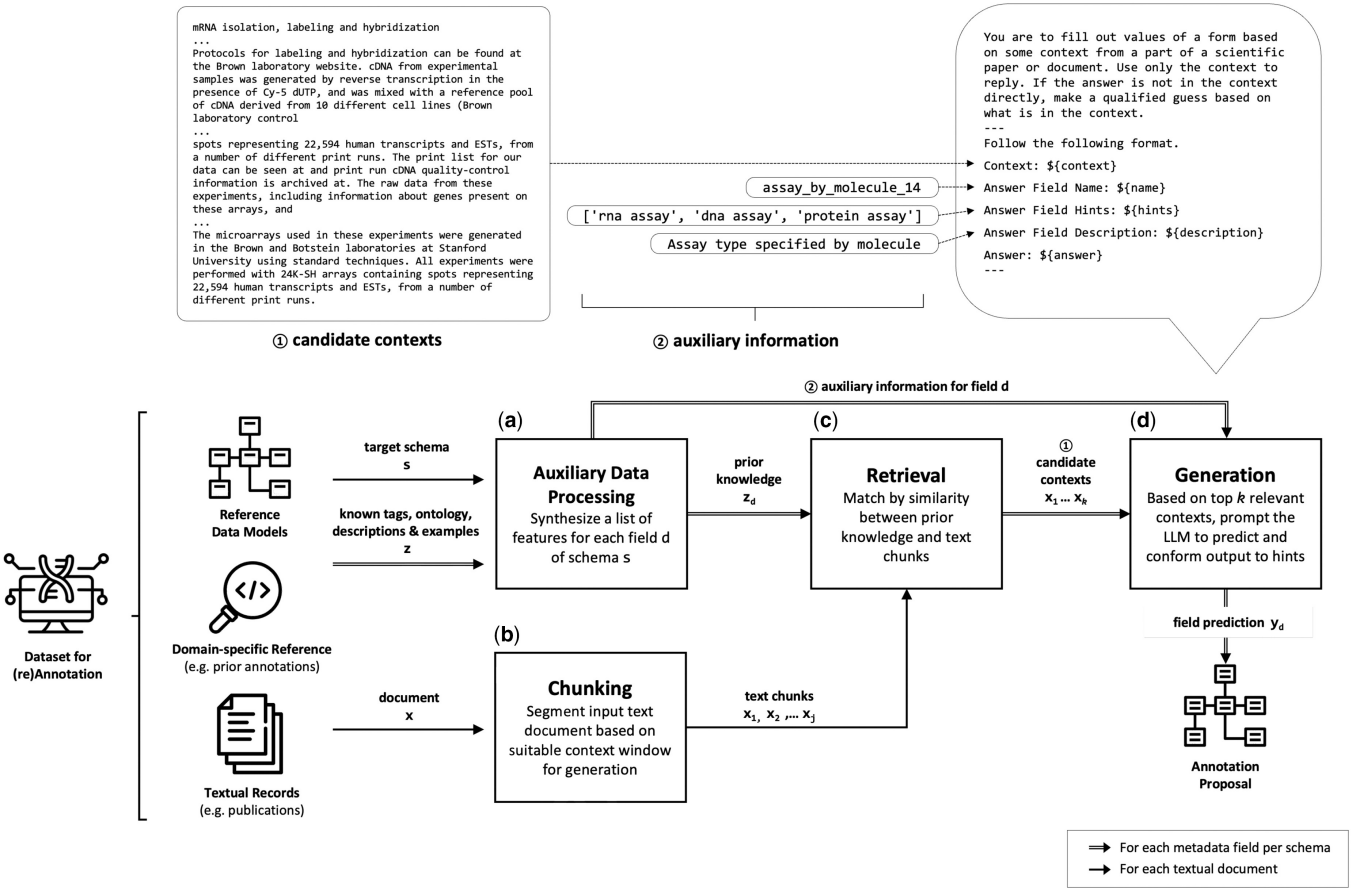
The Pre-Meta data annotation pipeline consists of four components: (a) an auxiliary data processing step that takes a schema and references related to a domain (e.g., pre-generated metadata tags, ontologies, etc) and outputs representations of the auxiliary data as useful prior knowledge to facilitate the retrieval process; (b) a chunking step that segments the input document into suitable chunk sizes; (c) a retrieval step that takes prior knowledge from the auxiliary data processor and text nodes from the chunking step, and outputs top *k* candidate contexts for prediction generation; (d) a generation module taking *k* contexts and auxiliary information as hints for generation, and applies a user-defined LLM to predict a metadata field.

The Pre-Meta data annotation pipeline contains two main parts: first, the auxiliary data processing step shown in Fig. 1a; second, a simplified RAG defined as Eq. (1) consisting of three blocks of processes illustrated in Fig. 1b–d: chunking, retrieval, and generation. Given a dataset of interest requiring (re)annotation, the dataset’s textual references are processed by a chunking step, which outputs chunks of text to the retrieval module. The retrieval module uses various representations of auxiliary information to identify *k* relevant contexts. The generation module, for each field of the target schema, proposes an annotation based on relevant contexts.

#### 2.2.1 Auxiliary data processing

For the task of data annotation, given a data schema of interest, we considered three types of auxiliary information: (1) a set of pre-generated metadata tags associated with each schema field which is known to the user or accessible through related instances of data annotations on existing data portals; (2) an ontology of the associated knowledge domain, with nodes similar to the expected values of the field, and (3) example values provided in the given schema.

The processor (Fig. 1a) takes the data schema in the form of a pydantic object which characterizes each metadata field as a Literal type accompanied by a Field description. We also follow this convention to format pre-generated metadata tags as a Literal list (e.g., Literal[’rna assay’, ’dna assay’, ’protein assay’] as shown in Fig. 1) for use in both retrieval and generation steps. For ontologies, we selected a node and used all its descendant nodes (i.e. the nodes in the subtree/subontology with the selected node as root node). To do that, we used the owlready2 package (Lamy 2017) to operate on each .owl file. As an example, for the field “assay by molecule”, we used the Internationalized Resource Identifier (IRI) code OBI_0000070 to locate the “assay” node object in the OBI ontology. Then we collated all descendant nodes for their descriptions without maintaining their tree structure. The resulting list of subontology information would then be passed onto the retriever for context ranking (Fig. 1c).

#### 2.2.2 Chunking

The chunker (Fig. 1b) loads full text of papers and textual documents in .xml format using UnstructuredXMLLooader (https://pypi.org/project/unstructured), which is then cleaned by removing noisy text headers and tags using LangChain’s MarkdownHeaderTexSplitter and split into smaller chunks, Chunks(x) = $\left\{x_{1},x_{2},\ldots ,x_{j}\right\}$, using the RecursiveCharacterTextSplitter (https://python.langchain.com). Finally, the resulting chunks are stored as LlamaIndex’s TextNode data object (Liu 2022).

#### 2.2.3 Retrieval

The retriever (Fig. 1c) embeds the document chunks and prior knowledge $z_{d}$ for the field using a sentence transformer (Reimers and Gurevych 2019) all-MiniLM-L6-v2 from Hugging Face, and compares these using cosine similarity to rank the chunks based on the relevance for the field. The retriever allows for a list of *n* queries, $z_{d}=z_{d}^{1},z_{d}^{2},\ldots z_{d}^{n}$, where each string $z_{d}^{i}$ is embedded separately. The string $z_{d}^{i}$ with maximum similarity to each chunk $x_{j}$ is used for the ranking:


(2) $$\text{relevance}\;\text{of}\;\text{chunk}\;x_{j}=\underset{i\leq n}{\max}(\text{cos}\_\text{sim}(\text{emb}(z_{d}^{i}),\text{emb}(x_{j})))$$

Top *k* chunks with highest similarity scores are concatenated in text as one candidate context and provided to the generation module through a prompt template (the “candidate context” part as shown in Fig. 1–1).

#### 2.2.4 Generation

Generation (Fig. 1d) is independent of LLM models. Each target metadata field is annotated by feeding the generation model a prompt following the template in Fig. 1, filled with the retrieved context and field property values (name, hints, and description). Constrained generation is used to ensure an output format in line with the field hints. This works by masking the probabilities of the tokens not leading to allowed tags given as hints, before sampling the next token, thereby always ending up with a compliant output. The output can be an integer, a string with possible length constraint, or, as in our experiments, one of several listed possible strings. To do this, we use the outlines package (Willard and Louf 2023) for open-weight models, and OpenAI’s native structured outputs for OpenAI models.

## 3 Experiments

### 3.1 Data schema selection

To experiment and evaluate a new dataset annotation method, a data schema with real-world metadata fields is preferred. In the experiment, metadata fields from the ArrayExpress portal were selected. ArrayExpress is one of the major public repositories for functional genomic datasets. For our experiment, we selected five fields shown in Table 1, based on field relevance and technical suitability. More specifically, the selected fields align strongly with a real-case scenario where a biologist or other biomedical researcher performs a dataset query, such as searching for a dataset concerning a particular tissue (ArrayExpress field: “organism part”) or experiment design like compound treatment or time series (ArrayExpress field: “experiment design”). In addition, they are highly descriptive and associable to existing ontologies, ensuring that they can be mapped to controlled values. Finally, selected fields from ArrayExpress are mapped to other public portals’ similar schemas for dataset access (e.g., GEO), underlying their importance for dataset annotation.

**Table 1.** A mapping of the evaluation metadata fields (Col. 1) to their respective relevance (Col. 2), their GEO analogous fields (Col. 3), and viable sample sizes of paper per field after filtering, and finally to their associative ontologies (Col. 5) that can be pruned to subsets of node descriptions (Col. 7) using specific entity terms (Col. 6).

		Pre-generated metadata		Ontologies		
Metadata field	Relevance	GEO mapping	Sample size (papers)	Ontology	Subtree top node	No. of nodes
hardware	Represents sequencing or/and microarray platform used to generate experimental data	“Platforms”	1526	OBI	“DNA sequencer”	37
organism part	Represents the specific biological sample origin, such as “liver”, “blood”, or “tumor tissue”	“Source name”	4773	BTO	“Tissues, cell types, and enzyme sources”	6451
experimental design	Captures the structure and purpose of the study (e.g., “time-course”, “case-control”, or “dose-response”)	“Overall design”	5647	EFO	“Study design”	64
study type	Represents a more concise description of the experimental process for the dataset generation; defines the method or approach used in the experiment (e.g., “RNA-seq”, “ChIP-seq”, “DNA methylation”)	“Experiment Type”	14797	EFO	“Assay”	556
assay by molecule	Describes the molecular target of the assay (e.g., “RNA”, “protein”, “DNA”)	“Extracted molecule”	14344	OBI	“Assay”	1656

Table 1 shows the selected fields of ArrayExpress for evaluation, including the counterpart of each field in GEO and the respective sample size in the test dataset. Although cBioPortal does not offer direct equivalents for several ArrayExpress metadata fields, relevant information can often be included in the description field of the meta_study.txt file or within custom attributes (e.g., Tissue_Site) in the clinical data files (data_clinical_sample.txt or data_clinical_patient.txt).

### 3.2 Data curation

To demonstrate the case of an automated data annotation pipeline, we need textual records associated with real-world biomedical datasets with the corresponding metadata that can be used as labels. We curated a dataset A=(X,Y) with 14 844 pairs of scientific papers and metadata files. Each pair (x,y)∈A consisted of *x*, an open access paper in .xml format from Europe PubMed Central (Europe PMC) and *y*, a set of labels that describe the associated dataset of the paper. The *y* is curated from the dataset’s metadata in .json format downloaded from ArrayExpress.

#### 3.2.1 Label standardization

Each metadata file *y* crawled from ArrayExpress contained a set of fields with varying quantities and consistencies of tag values. For example, some of the fields had several hundred or even thousands of unique tag values, many of which were semantically equivalent but syntactically different (e.g., “male and female” versus “males and females”) as a result of the lack of schema enforcement. We merged and discarded the low-occurrence metadata tags, standardizing to a dataset where each field had up to 25 different possible labels.

Each paper has values for a non-empty subset of fields and may have several values per field; but for the experiments in this article we used only fields with a single value for simplicity. The resulting sample size for each field is shown in column 4 of Table 1.

**Figure 2.**
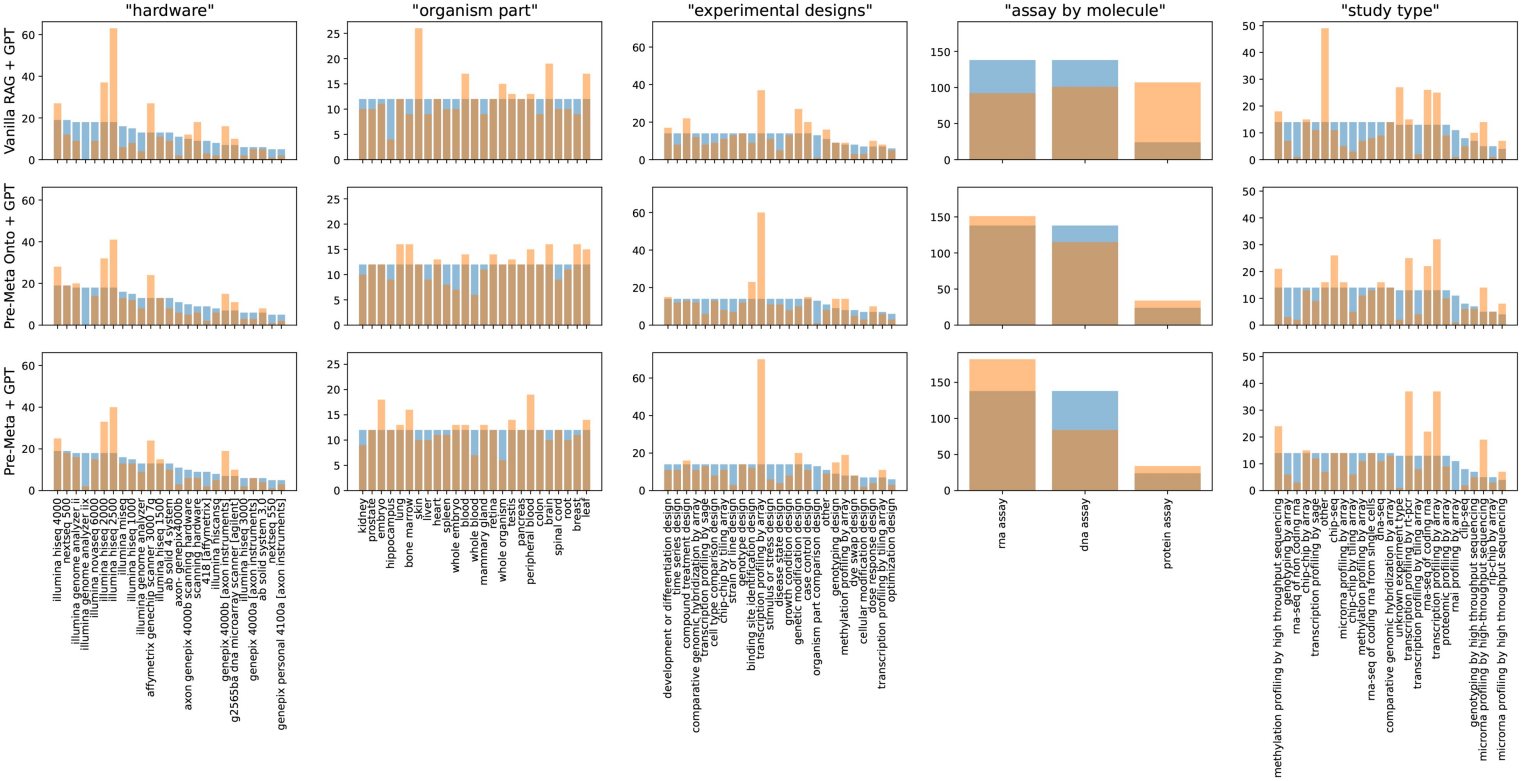
A comparison of the distributions of predicted metadata (orange bar) against actual annotations (blue bar) across five field types based on results from three GPT-4o mini pipelines (*Vanilla RAG*, *Pre-Meta Onto*, and *Pre-Meta*). Certain field labels, such as “illumina hiseq 2500” in hardware, “skin” in organism part, and “other” in study type show observable attenuation, while “transcription profiling by array” in experimental design shows further biased attention (*n* = 300).

The frequencies of the 25 possible labels were highly skewed, and for three of the fields, more than half of the papers had the same label. This leads to the potential for distributional “cheating”, where a model with a high chance of predicting these values could get a high score without using the context as intended. To combat this, a subset of 1500 pairs of paper and labels was sampled for use in the experiments, providing 300 samples for each field. The papers with the least frequent of the 25 possible labels were picked first, ensuring a uniform distribution of the labels as much as possible. The resulting label distribution for each field is shown as the blue bars in Fig. 2.

### 3.3 Design of experiment

In order to easily and effectively evaluate and compare different versions of the pipeline, we focus on the case with limited output space by using schema fields with a predefined set of possible values—essentially as a multiple-choice task. The alternative of using free-text fields would require a way of measuring the similarity of the output and the correct answer, for which embedding models are not necessarily accurate in representing specific biomedical terminologies. As the retrieval process is the part of the pipeline we focus on investigating in this work, different versions of it were tested. These versions, presented in Section 3.3.1, are based on using varying knowledge to represent the field. In the case of using ontologies as auxiliary information, we retain the allowable metadata tags as hints (Figs. 1 and 2) for the generation process in order to isolate the effects of the retrieval part, even though alternative retrieval methods are meant mainly for situations where this information is not known, such as working with open-string fields.

#### 3.3.1 Retrieval methods

The following list explains the different versions of the retrieval step of the pipeline that were used in the experiments. The first two are meant as baselines for comparison, while the rest are variants of Pre-Meta:

- *Fullpaper*: providing full text to GPT-4o mini without RAG and the use of auxiliary information.
- *Vanilla RAG*: using the original field description given by the target schema for ranking text chunks that represent candidate contexts.
- *Pre-Meta*: using individually encoded known metadata tags to rank chunks of candidate contexts using Eq. (2).
- *Pre-Meta Onto*: using individually encoded descriptions from the subtree of the related ontology to rank chunks of candidate contexts. The root nodes were chosen manually to include nodes similar to a substantial part of the known metadata tags, and are presented in Col. 5–7 of Table 1.
- *Pre-Meta (Retrieval-only)*: the retrieval-only case of Pre-Meta. Since a relevance score is calculated for each allowable metadata tag, it is possible to simply pick the one with the highest score as the prediction, skipping the generation step altogether.

#### 3.3.2 Generation

We evaluated different generation setups, including open-weight models (Mistral-7B-v0.1-GPTQ and Meta-Llama-3.1-8B-Instruct-GPTQ-INT4), proprietary GPT-4o mini, and one without a generation model for the retrieval-only case of Pre-Meta.

#### 3.3.3 Evaluation

For each paper, a score of 1 is given if the output of field prediction is the correct label and 0 otherwise. The reported scores are averages over the 300 papers for each field, and over five fields for each pipeline configuration. For fine-grain analysis and documentation, we recorded scores both field-by-field and as summary accuracy, together with all input RAG parameters and prompts using an AI developer platform, Weights & Biases (https://wandb.ai).

### 3.4 Results

Based on experimenting with 300 sampled paper-metadata pairs for each of five chosen fields, we observe considerable advantages in incorporating domain-specific auxiliary information for the task of predicting annotations for metadata fields. The results are illustrated as a summary of the accuracy scores in Table 2 and as distributions of predictions versus labels in Fig. 2. We observe that the Pre-Meta retrieval methods by far outperform conventional retrieval (*Vanilla RAG*), for pairing with both GPT-4o mini and open-weight models (Llama 8B and Mistral 7B). The accuracy gain is seen in all fields and Pre-Meta versions, with the exception of the “experimental design” field for GPT-4o mini, where conventional retrieval scored slightly better. In combination with GPT-4o mini, *Pre-Meta* and *Pre-Meta Onto* add 26% and 23% performance gain compared to the baseline of *Vanilla RAG*. The gain is especially pronounced for the pairing with Llama 8B and Mistral 7B, where *Pre-Meta* adds 72% and 75% to each respective model’s baseline performance. The *Pre-Meta (Retrieval-only)* setup, which involves directly choosing the best matching allowable metadata tags derived from auxiliary information without using a generation model, performs better than the baselines for the field “assay by molecule”, and is better on average than Mistral 7B with *Pre-Meta*. As this is a considerably cheaper method than the others, it shows that generation models alone do not always provide a better solution, at least for the tasks explored in this experiment.

**Table 2.** A ranking of combinations of retrieval methods and generation models based on their accuracy scores of annotation predictions averaged across five evaluation fields.

Retrieval method	Generation model	label: hardware	label: organism part	label: experimental design	label: assay by molecule	label: study type	Average accuracy
*Pre-Meta*	*GPT-4o mini*	*0.540*	*0.787*	*0.330*	*0.770*	*0.513*	0.588
*Pre-Meta Onto*	*GPT-4o mini*	*0.513*	*0.743*	*0.323*	*0.817*	*0.480*	*0.575*
Pre-Meta	Llama 8B	0.390	0.710	0.220	0.723	0.370	0.483
*Vanilla RAG*	*GPT-4o mini*	*0.323*	*0.673*	*0.343*	*0.627*	*0.367*	*0.467*
Pre-Meta Onto	Llama 8B	0.410	0.593	0.180	0.740	0.390	0.463
*Fullpaper*	*GPT-4o mini*	*0.340*	*0.730*	*0.160*	*0.683*	*0.270*	*0.437*
Pre-Meta	–	0.110	0.547	0.170	0.740	0.303	0.374
Pre-Meta	Mistral 7B	0.190	0.560	0.157	0.627	0.140	0.335
Pre-Meta Onto	Mistral 7B	0.167	0.413	0.093	0.643	0.123	0.288
Vanilla RAG	Llama 8B	0.170	0.483	0.123	0.490	0.140	0.281
Vanilla RAG	Mistral 7B	0.063	0.260	0.077	0.490	0.063	0.191

The rows in italic text illustrate that the combination of Pre-Meta retrieval (both variants) with GPT-4o mini as a generation model outperforms running the ***Fullpaper*** through GPT without RAG and running the GPT with ***Vanilla RAG***. Notably, both variants of Pre-Meta also add significant accuracy gain to Llama 8B and Mistral 7B compared to using conventional RAG. The pairings of both Pre-Meta methods with open-weight Llama 8B achieve comparable result to that of GPT-4o mini running on conventional RAG. Among GPT-4o mini pairings, ***Pre-Meta***contributes the highest accuracy to the fields of hardware, organism part, and study type, ***Pre-Meta Onto***to the field of assay by molecule, while ***Vanilla RAG***retains the highest on experimental design (*n* = 300).

Through improving the context ranking in retrieval alone, Pre-Meta methods add considerable gain in the prediction quality to our annotation tasks for the five selected fields, demonstrating the value of using pre-generated structured data as auxiliary information.

## 4 Discussion

### 4.1 Error analysis

#### 4.1.1 Domain-related

We note the high variance across five test fields. For example, all scores in “assay by molecule”, and almost all scores in “organism part”, are higher than every score in “experimental design”. This variability reflects the inherent heterogeneity of biomedical data and the uneven adoption of ontologies in existing repositories. For example, nuanced fields like “experimental design” remain challenging to annotate, as they require more comprehension of domain-specific contexts.

Figure 2 highlights the distribution of predictions versus labels across five field types for three different pipeline configurations. The distributions of predictions tend to match the labels better when using Pre-Meta, although certain field labels are systematically over- or under-represented.

Some labels of the organism part field are not mutually exclusive, e.g., “peripheral blood” and “whole blood” could be regarded as subcategories of “blood”. Thus, some errors can be attributable to imperfections in the dataset and evaluation method, not to the models. Theoretically, a merging of semantically similar tags in the dataset or evaluation process would result in a fairer score, but this would require a systematic analysis of the metadata tags that was considered outside the scope of this work.

#### 4.1.2 Model-related

Although our work focuses mainly on retrieval, we observe that the choice of generation models affects the resulting performance more than the choice of retrieval methods. From Table 2, we see that, regardless of retrieval methods, the GPT-4o-mini model’s overall scores are better than those of two open-weight models. Generation models are known to be sensitive to how the task is presented. The factor that turned out to play a considerable role in our experiment was the order of the list of hints given to the prompt. As a result, we shuffled the list each time to avoid systematic errors. Figure 4 shows that all generation models used in the experiment systematically focus more on answers that appear early among the list of hints provided to generation, even with a fairly short list of 25 answers. The bias is similar to the observed tendency that LLMs have in attending to information in the early and late parts of long context windows (https://github.com/gkamradt/LLMTest_NeedleInAHaystack). This order bias in token selection also provides us with a perspective beyond general tasks benchmarks (https://aimlapi.com/comparisons/llama-3-1-8b-vs-chatgpt-4o-mini) that reflect the difference between models (e.g., Llama 8B vs GPT-4o mini) used in the experiment.

#### 4.1.3 Retrieval by metadata tags versus ontologies

**Figure 3.**
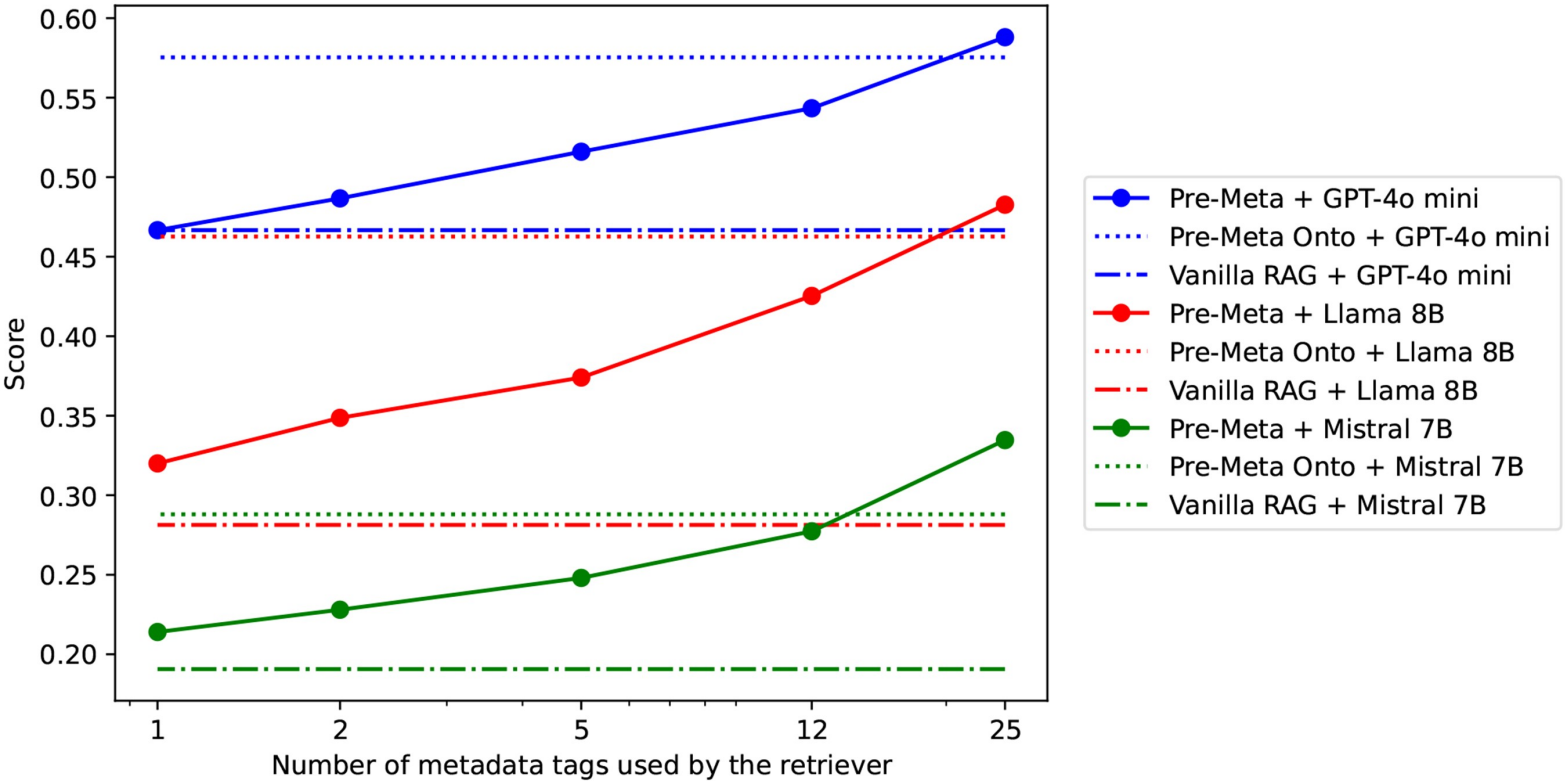
A comparison of the impact on annotation accuracy given different sampling densities applied to pre-generated metadata tags for ranking text chunks at the retrieval stage. For all paired models, we see comparable performance between using halved pre-generated metadata tags (12) and using ontology subtrees as auxiliary information. Notably, with full sampling at 25 metadata tags, the Llama 8B-paired accuracy (red solid) slightly exceeds that of GPT-4o mini on Vanilla RAG (blue mixed dash).

In instances where not all potential metadata tags are present, certain tags may still be available. To emulate this for *Pre-Meta*, we experimented with giving only a sampled subset of allowable metadata tags to the retriever as auxiliary information. The results of this sub-sampling are shown in Fig. 3 for comparison with *Pre-Meta Onto* pairings that use all available subontology field descriptions. We observe that even having a single metadata example given as a hint can yield superior retrieval quality compared to using just the field description itself in the *Vanilla RAG* baseline for Llama 8B, Mistral 7B; additionally, only two metadata examples are needed as a hint to bring improvement over the baseline for GPT-4o mini. Meanwhile, for all three paired models, the use of subontologies gives better results than using half or less than half of the available metadata tags.

**Figure 4.**
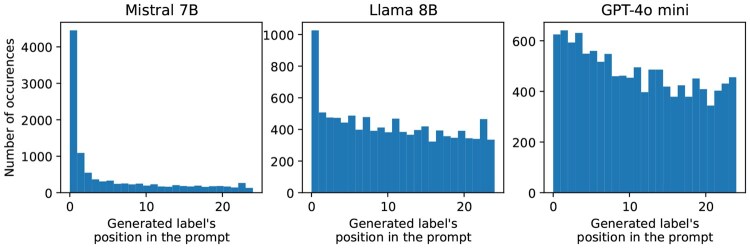
The graphs show how often an answer was generated, based on its position in the list of possible answers given in the generation prompt, across the fields with 25 possible answers. The list was shuffled at each LLM call. This shows that LLM models, especially Mistral 7B, have a systematic bias towards choosing an answer appearing early in the list (*n* = 10 800).

#### 4.1.4 Full context length versus chunks

Overall, *Vanilla RAG* performs better than *Fullpaper* by a slight margin (0.467 versus 0.437), although for only two of the five metadata fields. This shows some of the reason for using retrieval in the first place—having all the information in one large context window is not necessarily better than having a selection of small chunks of it, even when this selection is suboptimal (seeing as the Pre-Meta chunk selections are better).

### 4.2 Cost and computation

Table 3 itemizes the resource requirements across different usages of LLMs in the study. Compared to running on full paper, the use of GPT-4o mini on retrieved text chunks incurred significantly lower dollar costs due to lower token counts. For open-weight models running on a desktop GPU (Nvidia RTX 3090), the compute time of a few seconds per field also offers a promising option for deployment settings without access to non-industrial grade compute resources.

**Table 3.** Computational/monetary cost of experiments for each generation model.

Generation model	Cost per field	Cost of 300 papers with five fields
GPT-4o mini with retrieved chunks	0.0002 USD	0.3 USD/1 878 594 input tokens
GPT-4o mini with full paper	0.0027 USD	4.1 USD/26 992 061 input tokens
Llama 8B	3.5 s	88 min
Mistral 7B	3.9 s	98 min
Retrieval only	2.2 s	55 min

Computation times for Llama 8B and Mistral 7B includes just the generation step, not the document loading, retrieval and evaluation. Computation times are based on a single unit Nvidia RTX 3090 GPU.

### 4.3 Addressing FAIR principles

The experimental results demonstrate a promising step toward an LLM-driven automation of dataset (re)annotation. This effort provides the community with a useful building block to address the principle of machine-actionability (i.e. the capacity of computational systems to find, access, interoperate, and reuse data with none or minimal human intervention) emphasized in FAIR, in particular the characteristics stipulated in the subprinciples F2, F4, I1, and R1.3 (Relevant FAIR subprinciples are: F4. (Meta)data are registered or indexed in a searchable resource; F2. Data are described with rich metadata; I1. (Meta)data use a formal, accessible, shared, and broadly applicable language for knowledge representation; and R1.3. (Meta)data meet domain-relevant community standards. https://www.go-fair.org/fair-principles).

### 4.4 Related work

Efforts to increase the utility of LLMs in the biomedical domain include various Question Answer (QA) benchmarks such as BioASQ (Tsatsaronis *et al.* 2015), MedQA (D Jin *et al.* 2021), MMLU (Hendrycks *et al.* 2021), MedMCQA (Pal *et al.* 2022). There are also specialized pre-trained models like BioMedLM (Bolton *et al.* 2024) along with various fine-tuned, instruction-tuned, and Reinforcement Learning-tuned models seen on Open Medical-LLM Leaderboard (Pal *et al.* 2024), and more recently, pipelines driven by tool augmentations such as GeneGPT (Jin *et al.* 2024) that incorporate in-context learning through Web APIs of NCBI. These efforts, however, have not been extended to performing short-form answer generation tasks at the precision level suitable for metadata standardization.

Beacon V2 has been introduced as open-source software to facilitate the transformation of genomic metadata to conform to a hierarchical data model with seven entities: analysis, biosamples, cohorts, datasets, genomicVariations, individuals, and runs (Rueda *et al.* 2022). The input, notably, is an Excel file manually completed by the end user transcribing the metadata from the original storage format (e.g., text files, CSV, database, PEF, Electronic Health Records, etc) of the metadata. Regardless of the format, the origination of the metadata remains a user-defined process.

A potential prospect of automating the data profiling process can be seen in Extreme Multi-label Classification (XMC), a class of problem where a classifier selects relevant subsets of labels from an extremely large label set (Bhatia *et al.* 2016). XMC typically involves either fine-tuning a retriever (Decorte et al. 2023) over the label space or training a binary classifier per class (Clavié and Soulié 2023), both of which require substantial labeled data. To reduce manual labeling, researchers use distant supervision (Decorte *et al*. 2022), generate synthetic data with LLMs (Clavié and Soulié 2023, De Raedt *et al.* 2023), or fine-tune on related tasks with available data (Remy *et al.* 2022). At inference time, performance can be boosted by reranking candidate labels using LLMs. Zhu and Zamani (2023) avoid fine-tuning but rely on multiple LLM and retrieval calls per input to generate synthetic prompts and rerank labels. The Infer-Retriev-Rank method introduced by D’Oosterlinck *et al.* (2024) defined a multi-step, in-context interactions between LLMs and the retriever to predict relevant queries and interpret retrieved documents for better ranking. However, this requires dedicated prompt optimization for different datasets. Furthermore, the method focuses on the single task of predicting adverse drug reactions from an input document, which does not reflect the heterogeneity of metadata inherent in the rich description of genomic datasets. Pre-Meta, on the other hand, focuses on the setting of labeling diverse metadata fields through a complementary, light-weight procedure of leveraging available priors for candidate context ranking. Its contribution is also independent of performance gains that can be derived from fine-tuning, prompt optimization, and multiplying the use of LLM calls.

### 4.5 Limitations

Our current implementation focused on metadata that has string/literal values in order to leverage the LLM’s capabilities on natural languages. For other types of metadata, e.g., metadata with numerical values and alpha-numeric strings, additional experiments and iteration of the pipeline will be required, given the incoherent nature of the way numbers and decimals are tokenized by language models (Marjieh *et al.* 2025).

Due to the lack of existing reference or benchmark datasets for evaluating the tasks of biomedical data annotation, our choices of the five metadata fields were naturally constrained by the quality and availability of the metadata labels found on ArrayExpress. To further systematize the evaluation of Pre-Meta on a wider set and diversity of metadata fields that may appear under a different or newly introduced schema, additional data sources beyond ArrayExpress will need to be identified first and evaluated. Similarly, this applies to potential adoption of Pre-Meta to other domains. As part of further research, we aim to facilitate the automatic identification of relevant priors, i.e. from ontologies, that relate to specific new fields appearing through a new schema.

From the deployment perspective, the curation of data labels from pre-generated metadata tags in ArrayExpress required extensive cleaning due to the lack of schema enforcement. As a result, the raw state of metadata tags found in platforms like ArrayExpress would not be considered ready-to-go auxiliary information that can effectively support the implementation of Pre-Meta on the platform. But this gap can be addressed, portal by portal, through tailoring the auxiliary information preprocessor to reduce the data noise accordingly.

## 5 Conclusion

This study presents Pre-Meta, an LLM-agnostic and domain-independent data annotation pipeline designed to facilitate the automation of data profiling and downstream discovery. By leveraging auxiliary data—such as pre-generated metadata tags and ontologies—for retrieval to support the pairing with LLMs, Pre-Meta addresses key challenges in metadata standardization and dataset interoperability, as seen across repositories like GEO, ArrayExpress, and cBioPortal in the biomedical domain.

The scalability offered by LLMs in processing large datasets across repositories is both an opportunity and a challenge. While it enables the rapid annotation of diverse datasets, the reliance on unstructured or inconsistent metadata introduces variability in quality and interpretation (Higashi *et al.* 2024). Pre-Meta addresses this by promoting harmonization through auxiliary information and ontology-driven annotation. However, achieving full interoperability will require a broader community effort to establish harmonized metadata frameworks and robust cross-repository standards.

This study also emphasizes the need for benchmarkability in the evaluation of LLMs for data annotation in the biomedical domain. Transparent benchmarks and robust metrics are critical for assessing model performance across domain-relevant tasks, ensuring both accuracy and generalizability. Pre-Meta contributes to this goal by providing a scalable and flexible approach to metadata annotation, which lays the groundwork for improving data sharing and discovery in biomedical research.

## Data Availability

The code, data access, and pre-processing scripts are available in the GitHub repository: https://github.com/SINTEF-SE/LLMDap.
